# Observing Anthropogenic and Biogenic CO_2_ Emissions in Los Angeles Using a Dense Sensor Network

**DOI:** 10.1021/acs.est.4c11392

**Published:** 2025-02-13

**Authors:** Jinsol Kim, William M. Berelson, Nick Everett Rollins, Naomi G. Asimow, Catherine Newman, Ronald C. Cohen, John B. Miller, Brian C. McDonald, Jeff Peischl, Scott J. Lehman

**Affiliations:** † Department of Earth Science, University of Southern California, Los Angeles, California 90089, United States; § Department of Earth and Planetary Science, University of California, Berkeley, Berkeley, California 94720, United States; ∥ Department of Chemistry, University of California, Berkeley, Berkeley, California 94720, United States; ⊥ National Oceanic and Atmospheric Administration Global Monitoring Laboratory, Boulder, Colorado 80305, United States; # National Oceanic and Atmospheric Administration Chemical Sciences Laboratory, Boulder, Colorado 80305, United States; ∇ Cooperative Institute for Research in Environmental Sciences, University of Colorado Boulder, Boulder, Colorado 80309, United States; ○ Institute of Arctic and Alpine Research, University of Colorado Boulder, Boulder, Colorado 80309, United States

**Keywords:** greenhouse gas, emissions, fluxes, fossil fuel, biosphere, dense sensor network

## Abstract

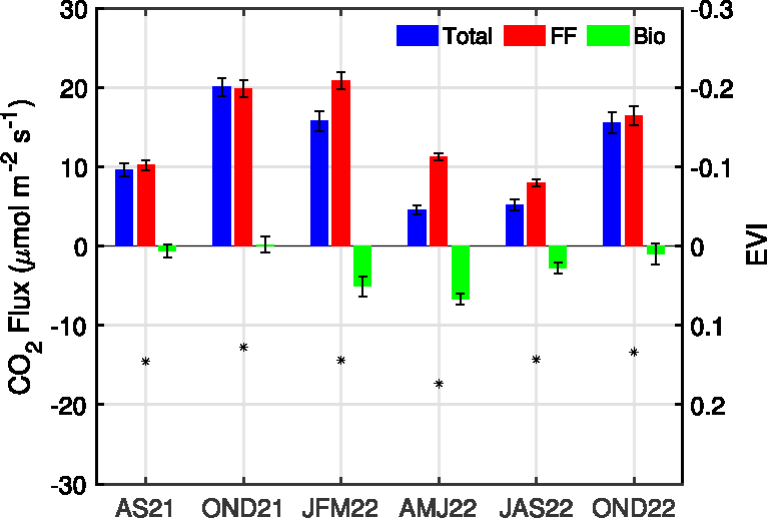

Urban areas are major contributors to greenhouse gas emissions, necessitating effective monitoring systems to evaluate mitigation strategies. A dense sensor network, such as the Berkeley Environmental Air-quality & CO_2_ Observation Network (BEACO_2_N), offers a unique opportunity to monitor urban emissions at high spatial resolution. Here, we describe a simple approach to quantifying urban emissions with sufficient precision to constrain seasonal and annual trends. Measurements from 12 BEACO_2_N sites in Los Angeles (called the USC Carbon Census) are analyzed within a box model framework. By combining CO_2_ and CO observations, we partition total CO_2_ emissions into fossil fuel and biogenic emissions. We infer temporal changes in biogenic emissions that correspond to the MODIS enhanced vegetation index (EVI) and show that net biogenic exchange can consume up to 60% of fossil fuel emissions in the growing season during daytime hours. While we use the first year of observations to describe seasonal variation, we demonstrate the feasibility of this approach to constrain annual and longer trends.

## Abstract 2

In this study, we investigate a simple method to quantify emissions using a high-density observation network. We show seasonal pattens in anthropogenic and biogenic CO_2_ emissions in Los Angeles. Additionally, we demonstrate the capability to effectively constrain annual emission trends.

## 1 Introduction

The Paris Agreement of the United Nations (UN) Framework Convention for Climate Change established an approach that signatory countries could take to reduce their greenhouse gas emissions and report the reductions publicly.^1^ In response, nations and cities worldwide are adopting mitigation strategies to reduce the level of CO_2_ emissions. These efforts are supported by collaboration through organizations such as the C40 Cities Climate Leadership Group (https://www.c40.org/) and the Global Covenant of Mayors for Climate and Energy (https://www.globalcovenantofmayors.org/), among many others. To support these urban efforts, the implementation of monitoring systems is crucial in evaluating and verifying the effectiveness of specific mitigation strategies in achieving the emission reduction targets specified by governments.

The current understanding of urban CO_2_ emissions relies most heavily on inventory-based methodologies. These “bottom-up” approaches include methods that estimate aggregate emissions in a domain using economic indicators, such as total fuel sales,^2^ and methods that provide more specific location and process information that rely on mapping the source-specific emission factors and measurements of activities,^3−5^ e.g., traffic patterns or average home heating use. In contrast, “top-down” approaches estimate emissions based on measurements of atmospheric CO_2_. Atmospheric transport modeling is necessary to interpret concentration measurements and solve the inverse problem. One approach involves using an inverse/data assimilation technique, optimizing the prior emission model. Both *in situ* and remote sensing observations have been used for top-down estimation.^6−12^ The majority of the studies using *in situ* measurements typically involve 2–15 observing sites within an urban region larger than 10 000 km^2^ equipped with state-of-the-art instruments that are calibrated frequently with gas standards.

The Berkeley Environmental Air-quality & CO_2_ Observation Network (BEACO_2_N) is designed to produce maps of urban air at high spatial resolution (2–4 km sensor spacing) while minimizing both capital and operating costs. Measurements of CO_2_, CO, NO_2_, NO, O_3_, and aerosols are provided using low-cost sensor technologies along with efficient methods for network scale calibration to keep labor costs low. Currently, the network consists of approximately 45 nodes in the San Francisco Bay Area, 12 nodes in Los Angeles, 20 nodes in Providence, RI, and 20 nodes in Glasgow, Scotland. The advantages of a dense network such as BEACO_2_N were evaluated using a hypothetical observing network and an inverse modeling system.^13^ The BEACO_2_N-like system, providing detailed maps of concentration variations within a city, outperformed conventional monitoring systems in effectively characterizing a point, line, or area source within an urban area. Turner et al.^9^ later used observations from an operating network combined with the inverse model to estimate total CO_2_ emissions and total CO_2_ reductions in a region of the San Francisco Bay Area before and during the COVID-19 shelter in place. They found an 8% reduction in emissions from stationary sources and a 48% reduction from traffic. Fitzmaurice et al.^14^ evaluated the capability of the inverse model to constrain the effect of vehicle speed and fleet composition on CO_2_ emissions. Asimow et al.^15^ reported a decrease in CO_2_ emissions at a rate of 1.8 ± 0.3% per year in the region based on nearly 5 years of observations.

In addition to these sophisticated and computationally intensive inverse modeling approaches, it is beneficial to consider simpler methods of analysis. For example, the use of BEACO_2_N observations to constrain policy-relevant trends in highway traffic emissions has been previously demonstrated using the correlation between the observed CO_2_ concentration and traffic flow rate.^16,17^ In this study, we explore a box model approach to quantifying total CO_2_ emissions within an 18 × 10 km section of Los Angeles. Measurements from a set of sites located along the prevailing wind direction are combined with meteorology information within a box model framework and then used to assess emissions in central Los Angeles (LA) where 12 BEACO_2_N nodes have been operating since June 2021 (called the USC Carbon Census). Anthropogenic and biogenic CO_2_ emissions are partitioned using constraints from observed carbon monoxide (CO) and assumptions that it is a proxy for fossil fuel CO_2_ (CO_2_ff). This approach assumes a time variable ratio between CO/CO_2_, which are co-emitted during combustion.^18−20^ While the CO to CO_2_ff emission ratio varies with the source allowing for some ambiguity, we use an additional constraint based on radiocarbon (^14^C)^19,21^ applied to atmospheric measurements in Los Angeles to narrow the range of plausible emission estimates. We treat the difference in CO_2_ff from net CO_2_ emissions as a measure of biogenic effects on CO_2_; the biosphere is both a source and a sink for urban CO_2_.

## 2 Methods

### 2.1 Measurements

**Figure 1:**
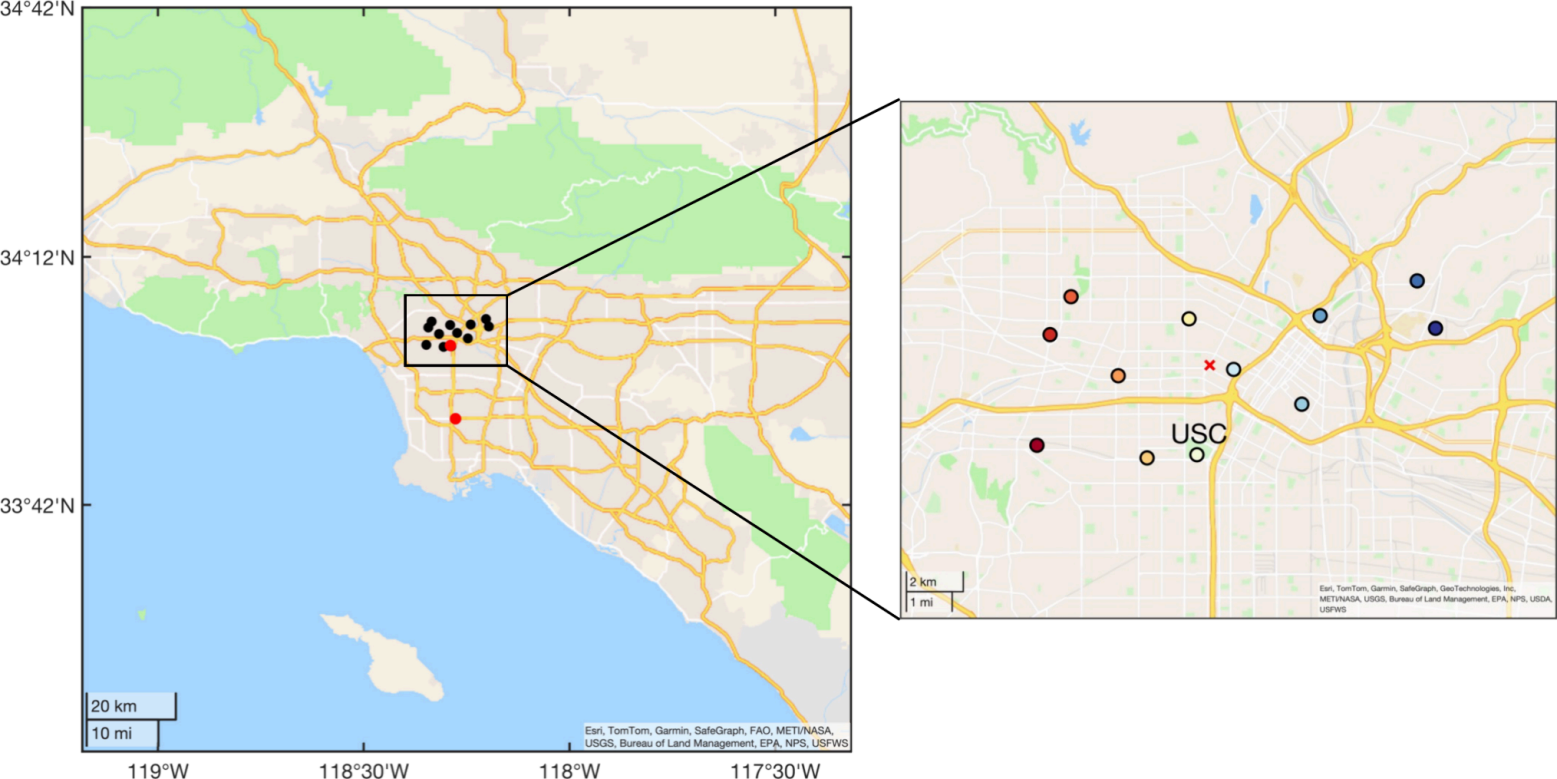
Map of Los Angeles showing BEACO_2_N-LA node locations (black circles on the left and color coded on the right) and the two Los Angeles Megacity Carbon Project sites used for calibration (red circles). The red × marker in the inserted map indicates the geographic center of the 12 nodes.

**Figure 2:**
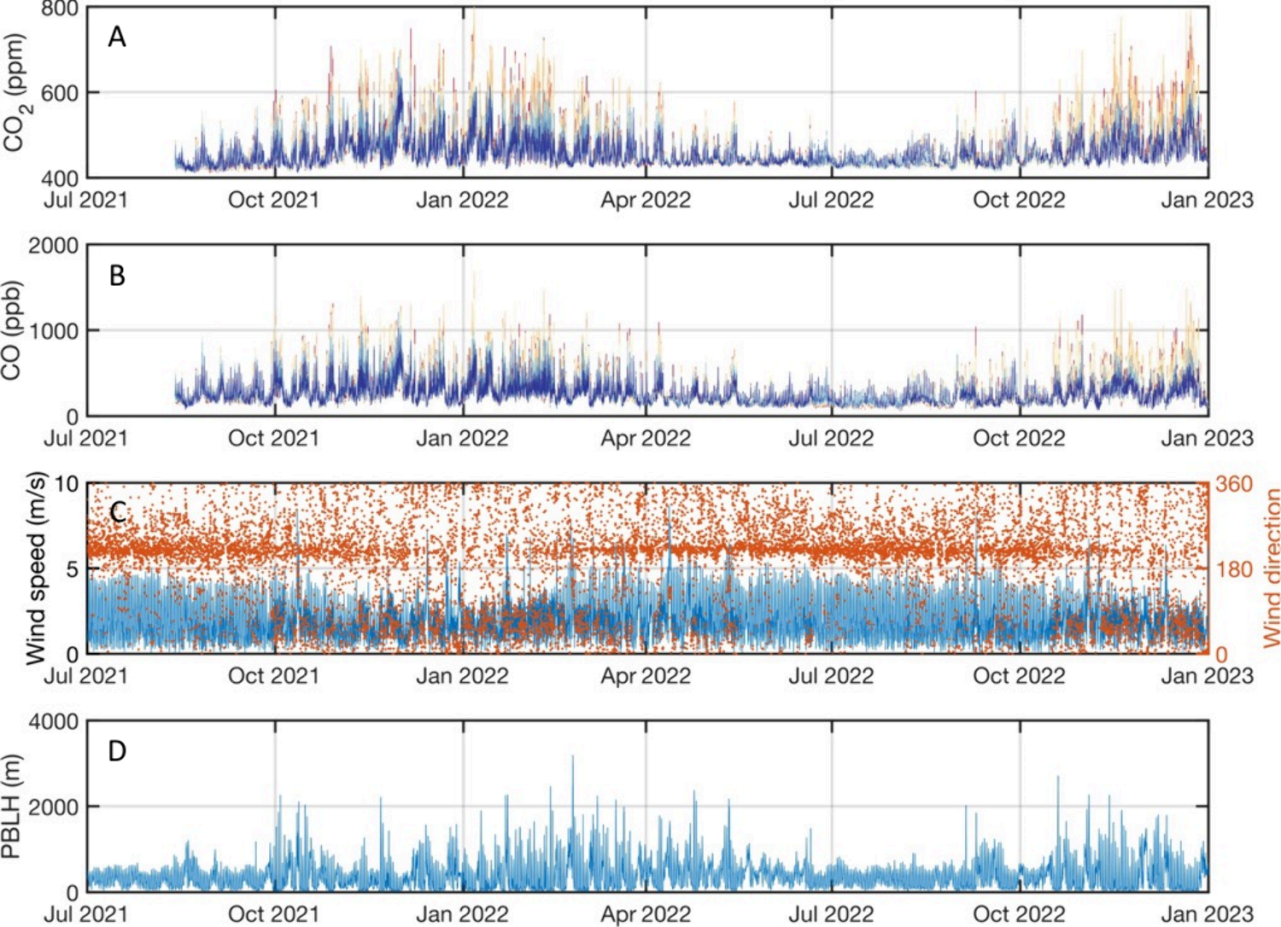
Observation of (a) CO_2_ and (b) CO used in this study from all USC Carbon Census sites. Different colors represent different sites corresponding to the colors in the inserted map in Figure 1. Network average (c) wind and (d) planetary boundary layer height (PBLH) from the HRRR model.

We use CO_2_ and CO measurements from a high-density observing system, the USC Carbon Census network, located in central LA (also known as BEACO_2_N-LA). A total of 12 nodes have been deployed on ∼4 km spacing (see Figure 1) beginning in June 2021. Observations from the USC Carbon Census network are supplemented by observations located on the University of Southern California (USC) campus, including measurements from a Picarro G2131i cavity ring-down spectroscopy (CRDS) instrument measuring ^12^CO_2_, ^13^CO_2_, and CH_4_ and the Los Angeles Megacity Carbon (LAMC) Project measurements at USC and Compton (COM) site, including Picarro G2301 (measuring CO_2_ and CH_4_) and Picarro G2401 (measuring CO_2_, CH_4_, and CO), respectively,^22,23^ for *in situ* field calibration. *In situ* field calibration involves comparing the background signal of each measurement to reference measurements with a precision of 0.1 ppm for CO_2_ and 5 ppb for CO. This process includes correcting sensitivity, bias, and drift and applying adjustments for temperature and humidity dependence. A detailed description of the design, deployment, and calibration of BEACO_2_N instruments can be found elsewhere.^24−27^ The precision of the hourly CO_2_ mole fractions is estimated to be ±0.5 ppm, and the accuracy is 1–2 ppm. The processed CO concentrations are estimated to have a precision of ∼100 ppb at an hourly resolution. We use the hourly averaged concentration of CO_2_ and CO between July 2021 and December 2022 (see Figure 2), which show large diurnal variation as well as seasonal variation. These fluctuations are associated with variations in emissions as well as meteorological conditions.

### 2.2 Box Model Approach for CO_2_ Emission Estimation

We use a box model approach based on the mass conservation as in the work of Strong et al.^28^ and Balashov et al.^29^
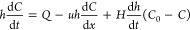
1The left-hand side of the equation represents the change in concentration *C* (μmol m^–3^) with time at sites within the compartment volume. The terms on the right-hand side of the equation represent emission (or uptake), advection, and entrainment, respectively. All terms in this model are given in flux units (μmol m^–2^ s^–1^). We assume the uniform emission inside the box at a rate of *Q* (μmol m^–2^ s^–1^) is well-mixed within a mixing layer with height *h* (m) and ventilated by winds blowing along the *x* axis with wind speed *u* (m s^–1^). When the mixing height is increased, the air above the mixed layer with concentration *C*_0_ (μmol m^–3^) is entrained into the box, which is represented with the Heaviside step function *H* that is *H* = 1 when d*h*/d*t* > 0 and *H* = 0 otherwise.

**Figure 3:**
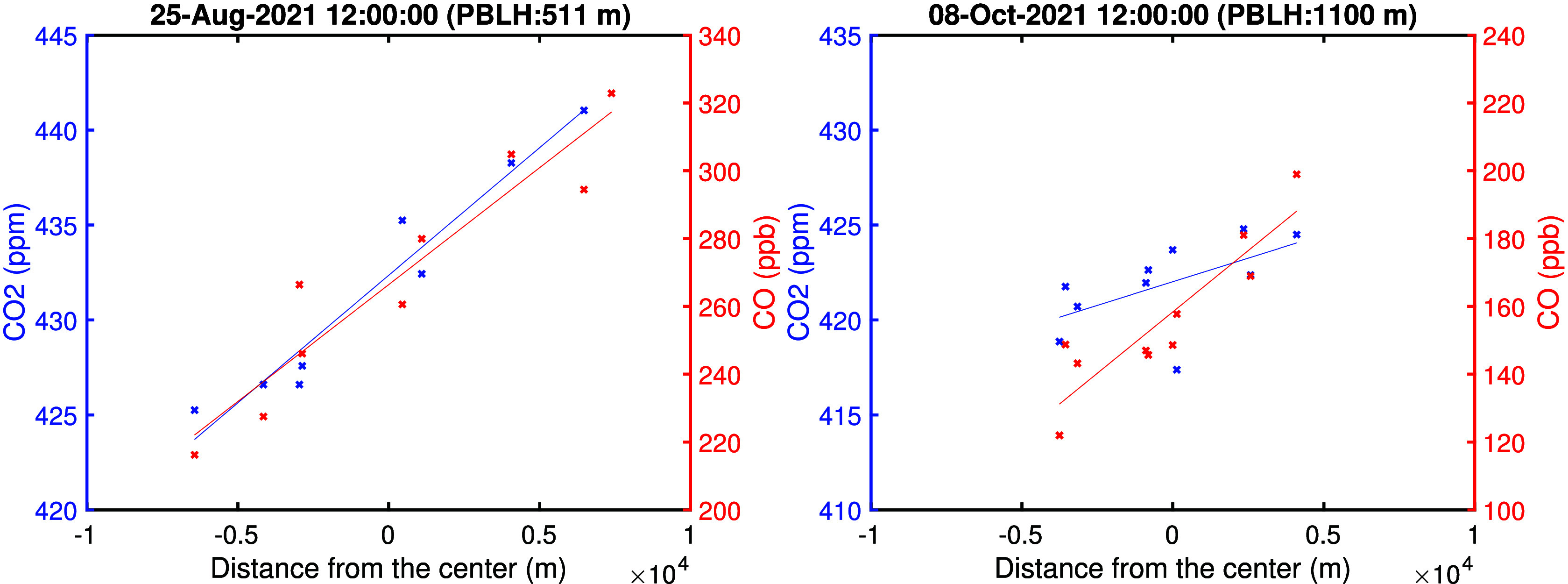
Example of a linear regression for calculating the term d*C*/d*x* at two different PBLH. CO_2_ is shown on the left axis, and CO is shown on the right axis.

To estimate emissions, *Q*, we rearrange eq 1 and apply it to hourly observations.
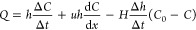
2Each term on the right-hand side of the equation is first calculated for each site and then averaged across the network. The change in concentration, Δ*C*, and the change in mixing height, Δ*h*, is calculated for each time step Δ*t* = 3600 s. The term d*C*/d*x* is calculated by leveraging the detailed mapping of the dense sensor network. Figure 3 shows an example of how d*C*/d*x* is calculated by combining all USC Carbon Census sites. For each time step, the *x* axis rotates along the wind direction, while the origin is fixed to the geographic center of the sites (red marker in Figure 1). When more than eight sites are available, observations from all available sites are projected onto the *x* axis (by drawing a perpendicular line to the wind axis). All concentrations measured from the network are compared, removing outliers that fall beyond ±2 standard deviations from the network median for each time step, and then d*C*/d*x* is calculated. The criteria of eight sites was chosen to include a significant portion (2/3) of the total domain of interest. This expands the time available for analysis to include the period before completion of the full sensor deployment. Entrainment is significant during morning when the mixing height is increasing, and the residual layer is mixed into the planetary boundary layer. The concentration, *C*_0_, of the residual layer is defined as the concentration from the previous day at 2 PM when the mixing height is generally at a maximum and before nocturnal boundary layer starts to form. Estimates of *h* and *u* are taken from the National Oceanic and Atmospheric Administration (NOAA) High Resolution Rapid Refresh (HRRR) for each site.

### 2.3 Partitioning Fossil and Biogenic CO_2_ Emissions

Carbon monoxide (CO) is a widely used tracer to estimate fossil fuel emissions as CO is often co-emitted with fossil fuel CO_2_ (CO_2_ff) during incomplete combustion.^10,20,30−32^ If the COxs/CO_2_ff ratio (*R*_ff_, where COxs is the CO enhancement above the background) is well-constrained, continuous CO measurements combined with *R*_ff_ can provide an estimate of continuous CO_2_ff. CO also has some contribution from oxidation of methane and volatile organic compounds (VOCs), and its oxidation can serve as a sink. However, the transport time across the study domain is short enough (less than 2 h) that removal of CO and methane oxidation can be ignored.^33^ A previous study also showed that VOC oxidation provides less than 1% of observed CO in a heavily polluted region, such as the LA region.^34^

We first calculate net CO and CO_2_ emissions, *Q*_CO_2__ and *Q*_CO_, using the procedure described in section 2.2. The net CO_2_ emissions are the sum of fossil and biogenic terms. To isolate the fossil fuel term, *Q*_CO_2_ff_ is estimated by assuming fossil CO_2_ is proportional to the CO emissions with a proportionality constant of 1/*R*_ff_.
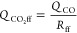
3We then estimate biogenic CO_2_ emissions (*Q*_CO_2_bio_) as the difference between the total CO_2_ emissions (*Q*_CO_2__) and fossil fuel CO_2_.
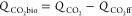
4Time steps with *Q*_CO_2_ff_ < 0 are physically unreal and were excluded as they indicate either a large error in meteorology data or meteorological conditions deviating from the condition assumed for a box model approach to be valid. Outliers of emissions beyond ±3 standard deviations from the mean within a 3 month moving window, which accounts for ∼2% of the hourly emission estimates, were also excluded.

We estimate *R*_ff_ from bottom-up inventories. The 2021 values were not available at the time of the writing of this manuscript. First, we use 2019 annual CO_2_ emissions and CO emissions in Los Angeles County for each source sector from Vulcan 3.0 and the California Air Resources Board (CARB) California Emissions Projection Analysis Model (CEPAM). The 2015 CO_2_ emissions in Vulcan 3.0 are scaled by the emissions in the CARB greenhouse gas emission inventory data to estimate 2019 emissions. We opt to use the 2019 estimate due to the exceptional circumstances of reduced emissions during the pandemic in 2020. The ratio of CO_2_ emissions and CO emissions is calculated for each source sector (*R*_*x*_; see Table S1 of the Supporting Information), which we assume constant over our study period. We combine constant *R*_*x*_ estimated from bottom-up inventories and sector partitioning information (relative contribution of each source sector, *f*_*x*_) that varies in time collected from Hestia-LA at hourly resolution to estimate *R*_ff_ following Kim et al., which has been evaluated against ^14^C data collected over a year long period in 2015.
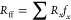
5For 2 km circles around each BEACO_2_N sensor, we average Hestia-LA sectoral emissions provided at 1 km spatial resolution.

**Figure 4:**
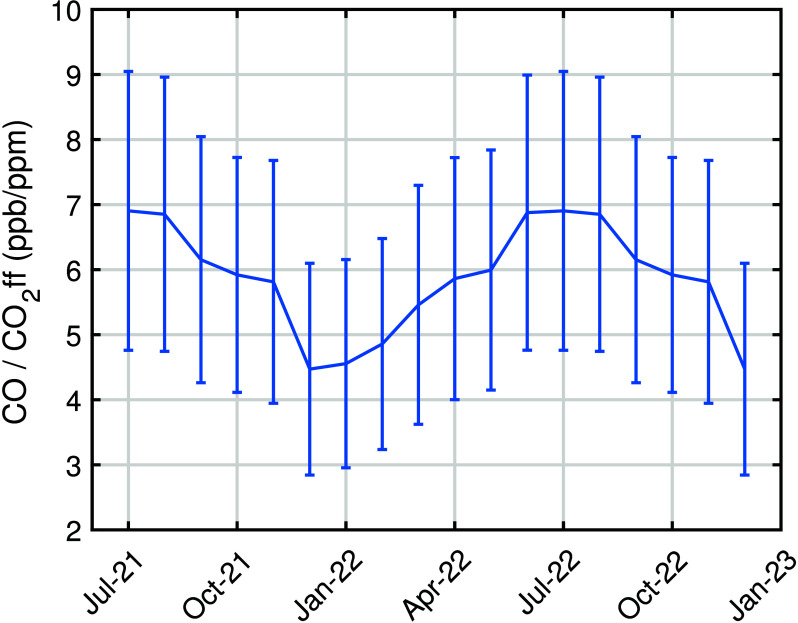
Monthly bottom-up inventory-based estimates of the CO to CO_2_ff emission ratio (*R*_ff_) adjusted by a scaling factor of 0.5. These values are used in eq 3 to calculate CO_2_ff emissions from CO.

We adjust the estimated *R*_ff_ value based on measurements made with flask air collected daily at 2 PM (LT) during the month long Southwest Urban NO_2_ and VOC Experiment in LA (also known as the SUNVEx-LA campaign, August 2021, https://csl.noaa.gov/projects/sunvex/), a NOAA-led experiment measuring various air pollutants. This campaign found *R*_ff_ of 4.2 ± 0.9 ppb ppm^–1^ (mean and standard deviation) determined from ^14^C and CO measurements of flask air samples (see the Supporting Information). Measurements made after August 21st, 2021, were excluded due to the impact of wildfires on observations. We again combine *R* and the relative contribution of each source sector following the approach presented in eq 5. Hestia-LA is weighted and averaged by the footprints (ppm per μmol m^–2^ s^–1^) of each grid (see section 2.4 for additional details on footprints). Our bottom-up inventory-based estimate of *R*_ff_ for August 2021 is 8.5 ± 2.5 ppb ppm^–1^ (mean and standard deviation), which is larger than the value from the flask measurements. It is likely that the overestimation of *R*_ff_ from the bottom-up inventory is driven by the error in *f*_*x*_ due to the possibility of a change in sector partitioning from 2015. We multiply a scaling factor of 0.5 ± 0.2 to the bottom-up inventory-based *R*_ff_ estimates for our study domain. We use monthly averaged corrected *R*_ff_ (Figure 4) and eq 3 to produce estimates of fossil fuel CO_2_ flux. Note that emissions from biofuel and human respiration are included in the biogenic sources in this study that would lead to an underestimation of fossil fuel emissions. Miller et al.^35^ estimates biofuel emissions to be 10% of fossil fuel emissions in Los Angeles basin. Lower *R*_ff_ in winter is driven by increased emission in residential and commercial sectors, which have low *R* values.

### 2.4 Synthetic Data Experiment

We used a synthetic data experiment to evaluate the box model approach. Synthetic observations of the USC Carbon Census network are generated from July 2021 to July 2022 using the Stochastic Time-Inverted Lagrangian Transport (STILT)^36,37^ model combined with the meteorological fields from HRRR. The STILT model is an atmospheric transport model frequently used in inverse modeling approaches that computes footprints indicating the receptor’s sensitivity to surface emissions. The convolution of footprints (ppm per μmol m^–2^ s^–1^) and fluxes (μmol m^–2^ s^–1^) yields the synthetic enhancement (ppm) above the background. We add the background, estimated from Los Angeles Megacity Carbon (LAMC) Project as described by Verhulst et al.,^22^ to yield synthetic observations (ppm). We use a high-resolution fossil fuel emission product, Hestia-LA,^4^ for CO_2_ fluxes and Hestia-LA multiplied by a bottom-up inventory-based estimate of *R*_ff_ at hourly resolution (see section 2.3) for CO fluxes. A comparison between the observed CO_2_ (CO) and simulated CO_2_ (CO) is shown in Figure S2 of the Supporting Information. Lastly, we apply the box model approach to the generated synthetic observations quantifying the flux estimates and evaluate it against the model reference flux. The modeled reference flux is defined as the Hestia-LA emission rate averaged over 2 km circles around each sensor, which should incorporate a significant portion of the regions located between the sites.

## 3 Results and Discussion

### 3.1 Synthetic Data Experiment To Determine the Effective Mixing Height

While the box model approach assumes that the emitted gases mix throughout the entire planetary boundary layer (PBL), previous studies have shown that this is not realistic in urban environments where strong sources exist in the near field of measurement sites.^8,38^ To address this issue, we determined the effective mixing height from the synthetic data experiment. We use an effective mixing height for *h* in eq 2 varying between 0.1*h*_HRRR_ and 1.0*h*_HRRR_, where *h*_HRRR_ is PBL height estimates from HRRR, and evaluate the estimated flux compared to the modeled reference flux from Hestia-LA inputs.

**Figure 5:**
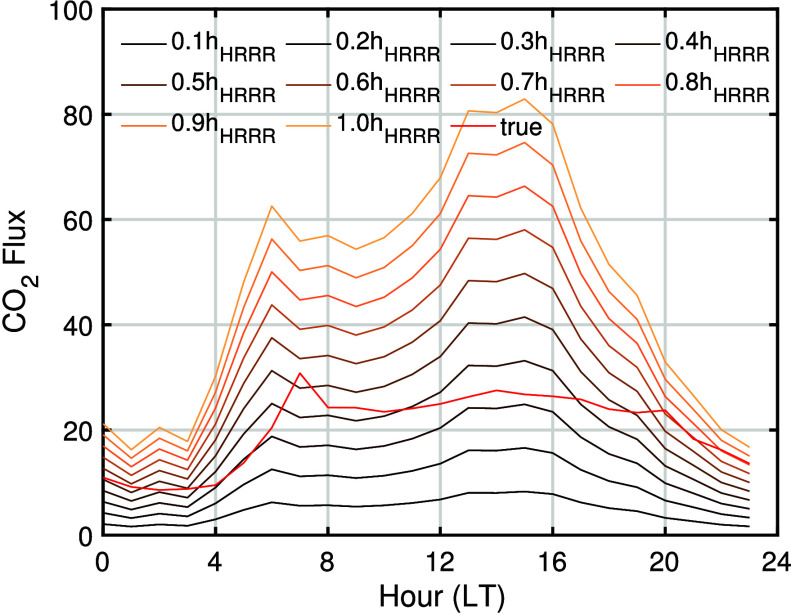
Diurnal pattern of fossil fuel CO_2_ fluxes estimated from synthetic observation between July 2021 and July 2022 using the effective mixing height varying between 0.1*h*_HRRR_ and 1.0*h*_HRRR_, where *h*_HRRR_ is PBL height estimates from HRRR. The red line represents the reference flux from Hestia-LA.

Figure 5 shows the diurnal pattern of estimated fluxes calculated using various effective mixing heights. We find that estimated daytime fluxes show reasonable agreement at *h* = 0.3 – 0.4*h*_HRRR_, but nighttime fluxes are always underestimated by our model. During the day, using a low effective mixing height results in underestimation of the flux and using a high effective mixing height results in overestimation of the flux. We estimate the effective mixing height in the location of the USC Carbon Census network to be 0.4*h*_HRRR_ and then use this value to estimate the flux for the daytime hours (from 1100 to 1700 LT) in the following sections 3.2 and 3.3. This process of determining effective mixing height is also feasible with publicly available coarser emission inventories or a simply constructed emission inventory in the absence of a high-resolution fossil fuel emission product. For example, we derived the same effective mixing height of 0.4*h*_HRRR_ using a uniform emission rate across the LA basin (details provided in section S2 of the Supporting Information). We focus on the daytime hours when the atmosphere is closest to well-mixed and the bias in the meteorological model boundary layer height, which would propagate to the effective mixing height, is lowest. This is consistent with previous studies that use an inverse/data assimilation technique combined with meteorological models.

### 3.2 Synthetic Data Experiment for Uncertainty Assessment

We use this synthetic data experiment to evaluate the uncertainty caused by the various assumptions made in the box model approach. We also propagate the uncertainty in sensor observations (*C*), concentration above the mixed layer (*C*_0_), mixing height (*h*), and wind speed (*u*), wind direction by adding randomly generated noise in the Gaussian distribution for each hourly timestamp. The wind direction affects the analysis, as we rotate the *x* axis along the wind direction and calculate d*C*/d*x*. The uncertainty in the CO_2_ measurements is ±0.5 ppm, and the uncertainty in the CO sensor is ±100 ppb (see section 2.1). The uncertainty in *C*_0_ is estimated as the standard deviation of the difference between the background estimated from LAMC and the background estimated from the synthetic concentration from the previous day at 2 PM, as described in section 2.2: ±18 ppm for CO_2_ and ±105 ppb for CO. Uncertainty in meteorological data is from Verreyken et al.:^200^ ±250 m for mixing height, ±2.1 m s^–1^ for wind speed, and ±63° for wind direction. To estimate the uncertainty in CO_2_ff fluxes from CO, the uncertainty in monthly averaged *R*_ff_ of ±2.5 (standard deviation of bottom-up inventory-based hourly *R*_ff_) and the uncertainty in the scaling factor of ±0.2 (see section 2.3) is propagated.

**Figure 6:**
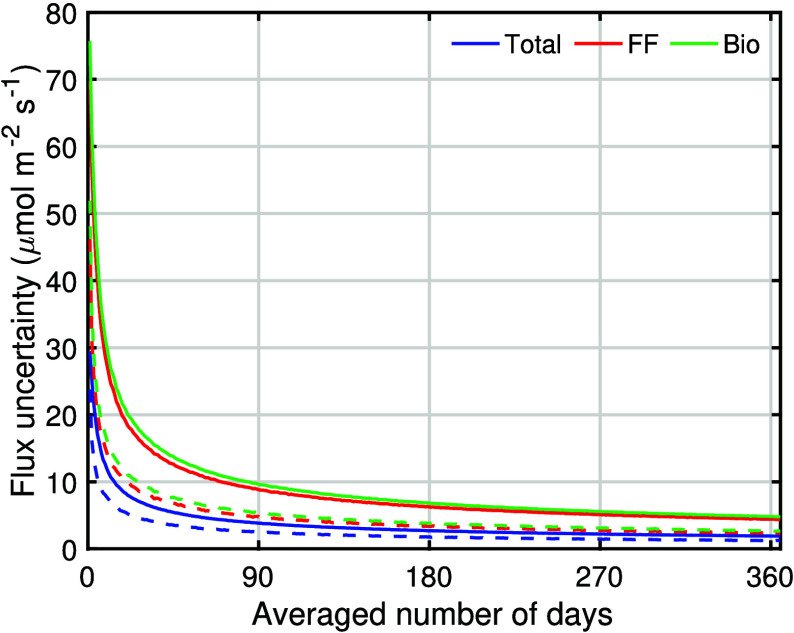
Uncertainty in flux estimates as a function of the number of days averaged using the bootstrap sampling method. The solid line indicates uncertainty in the hourly resolution data set, and the dashed line indicates uncertainty in the daily resolution data set averaged for each day using daytime hours (from 1100 to 1700 LT).

Table 1 shows the uncertainty in annual daytime fluxes for total CO_2_ and CO_2_ff. Uncertainty in estimated flux is calculated as the standard deviation of the difference between the estimated flux and Hestia-LA reference flux from 5000 bootstrap samples. First, daily daytime average flux is calculated and then averaged over randomly sampled 365 data points with replacement for each bootstrap sample. Not surprisingly, we find that uncertainty caused by the various assumptions made in the box model approach are a major factor in the total uncertainty, followed by the uncertainty in the wind data. For CO_2_ff fluxes, the uncertainty in the CO observation has a significant impact on the total uncertainty as well as the uncertainty in monthly averaged corrected *R*_ff_ as indicated by the difference between the uncertainty in the CO_2_ff fluxes and the uncertainty in the CO_2_ fluxes when the box model approach is the only uncertainty term. The total uncertainty in annual hourly fluxes is ±1.9 μmol m^–2^ s^–1^ for total CO_2_ and ±4.4 μmol m^–2^ s^–1^ for CO_2_ff. For annual daytime fluxes, the total uncertainty is ±1.2 μmol m^–2^ s^–1^ for CO_2_ and ±2.4 μmol m^–2^ s^–1^ for CO_2_ff. Then, the uncertainty in CO_2_bio fluxes is estimated to be ±4.8 and ±2.7 μmol m^–2^ s^–1^ for annual hourly fluxes and annual daytime fluxes. Hourly fluxes and daytime fluxes averaged for various time scales are shown in Figure 6. We find the total uncertainty in estimated CO_2_ff fluxes decreasing with a greater number of days averaged: ±8.2 μmol m^–2^ s^–1^ (21%) for monthly daytime average, ±4.8 μmol m^–2^ s^–1^ (12%) for seasonal daytime average, and ±2.4 μmol m^–2^ s^–1^ (6%) for annual daytime average. Recent studies have observed CO_2_ emissions decreasing at a rate of 2%/year.^15,39^ If a similar trend of decrease was occurring in Los Angeles, it could be observed within 3 years using this box model approach.

**Table 1:** Uncertainty in Annual Daytime Flux Estimates for Various Included Uncertainty Terms^a^

	flux uncertainty (μmol m^–2^ s^–1^)	
included uncertainty terms	CO_2_	CO_2_ff
box model	0.7	1.5
box model + sensor	0.7	2.1
box model + background	0.7	1.5
box model + PBLH	0.7	1.5
box model + wind speed	1.0	1.7
box model + wind direction	0.9	1.5
box model + all	1.2	2.4

a Flux uncertainty is calculated using bootstrap sampling, comparing the estimated flux to the true flux. First, daily daytime average flux is calculated and then averaged over 365 samples with replacement for each iteration. Note that the uncertainty in CO_2_ff also includes the uncertainty in monthly averaged corrected *R*_ff_.

### 3.3 Analysis of USC Carbon Census Network Data To Constrain Anthropogenic and Biogenic CO_2_ Emissions in Los Angeles

**Figure 7:**
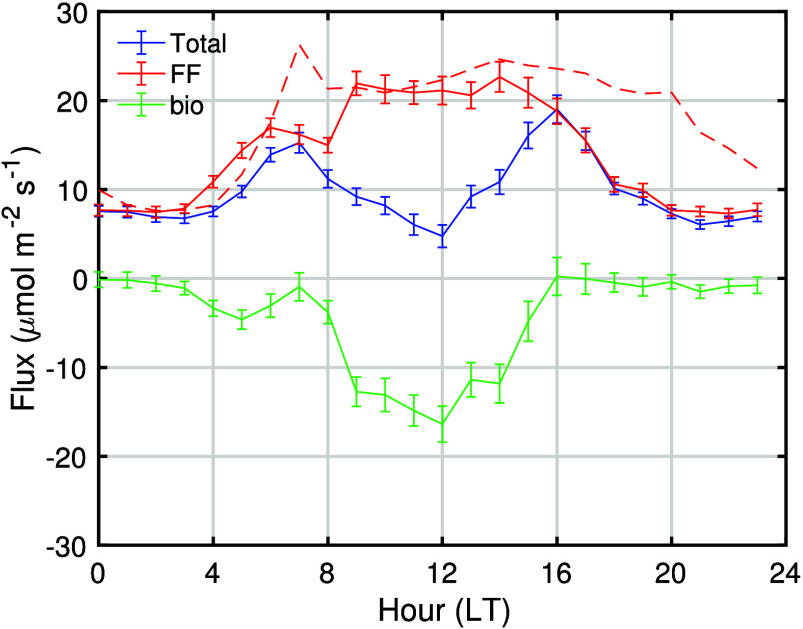
Diurnal variation of total, fossil fuel (FF), and biogenic (bio) CO_2_ fluxes averaged between July 2021 and July 2022. The error bars represent the confidence interval of each averaged values. The dashed red line shows diurnal variation in Hestia-LA 2015 emissions adjusted scaling to the CARB greenhouse gas emission inventory data to estimate 2022 emissions.

**Figure 8:**
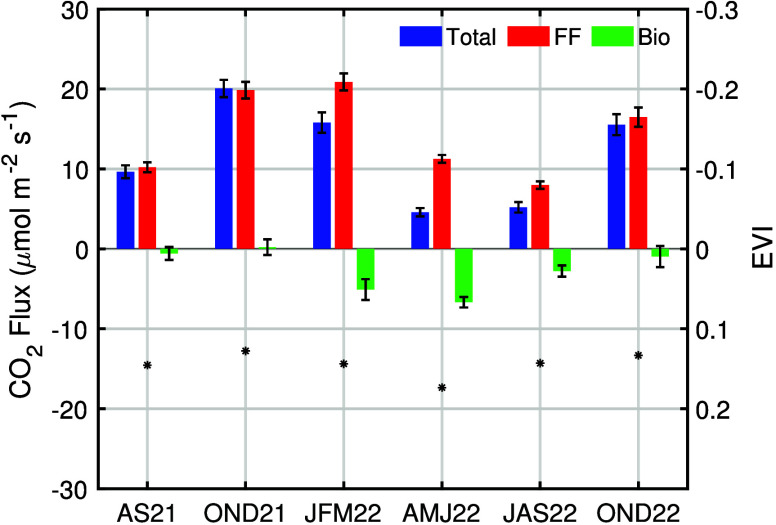
Seasonal variation of fossil fuel and biogenic CO_2_ fluxes during the daytime (from 1100–1700 LT). The error bars represent the standard errors of seasonal daytime fluxes. MODIS enhanced vegetation index (EVI) is shown reversed on the right axis to highlight its relationship with the biogenic flux uptake from the atmosphere (negative flux is maximum uptake).

Figure 7 shows the diurnal cycle of the estimated total CO_2_ fluxes and partitioned fossil fuel and biogenic fluxes averaged over an entire year at each time of day. Fossil fuel CO_2_ fluxes show a relatively smooth rise and fall over the course of the day, while pronounced biogenic uptake during daylight hours results in a total (net) CO_2_ flux that peaks early and late in the day. When the average is taken throughout the entire day, net biogenic CO_2_ uptake is considerable in this part of Los Angeles; biogenic uptake accounts for net sequestration of 4.5 ± 1.4 μmol m^–2^ s^–1^, equivalent to ∼30 ± 10% of the estimated fossil fuel emission flux of 14.1 ± 1.1 μmol m^–2^ s^–1^. Note that the errors reported in this section and shown in Figures 7 and 8 represent the 68% confidence interval of the averaged values, distinct from the hourly uncertainty estimated in section 3.2.

Figure 8 shows the seasonal variation in the derived daytime CO_2_ emissions for July to September (JAS) 2021, October to December (OND) 2021, January to March (JFM) 2022, and April to June (AMJ) 2022, respectively. Seasonal fluxes are calculated using the data when the wind is blowing from the southwest (dominant wind direction; see Figure 2) for a constant footprint, which represents the region for which our derived emission rate from the USC Carbon Census network is applicable. We observe higher fossil fuel emissions during January to June compared to July to December. This pattern can be associated with larger usage of natural gas for heating in winter.^40−42^ However, it can also be associated with the misrepresentation of meteorology in the model information. For example, Yadav et al.^43^ also observed a decreasing trend in emissions during summer months. In their study, this trend was attributed to large errors in wind speed that are generally lower in winter and often overestimated in the models.

Estimated biogenic fluxes (Figure 8) are consistent with the seasonality observed in the enhanced vegetation index (EVI), which serves as a measure of canopy greenness and is used as a proxy in biogenic models to estimate carbon uptake. EVI is averaged over 2 km circles around each sensor from a Moderate Resolution Imaging Spectroradiometer (MODIS) MCD43A4 Version 6 Nadir Bidirectional Reflectance Distribution Function-Adjusted Reflectance (NBAR) data set at 500 m resolution, at daily resolution representing 16 day moving averages.^44^ The low spatial resolution of the MODIS EVI likely diminishes its sensitivity to urban vegetation, and the fact that observations can only be made under clear sky conditions restricts its use for short-time scale analysis. However, we observe the expected seasonal variation in the raw data set, and we use the seasonal average to calculate the correlation coefficient. We observe maximum biogenic uptake of −6.7 ± 0.7 μmol m^–2^ s^–1^ in AMJ 2022 and maximum emission of 0.2 ± 1.0 μmol m^–2^ s^–1^ in OND 2021/2022, which corresponds to the inverse pattern in EVI (*r*^2^ = 0.7). EVI in Figure 8, which corresponds to the right axis, is shown in reverse. Note that AS 2021 only includes data from mid-August to September by the criteria to include more than eight sites; this could account for the difference between 2021 and 2022. We find that, during the daytime, the biosphere can consume up to 60 ± 6% of fossil fuel emissions of 11.3 ± 0.5 μmol m^–2^ s^–1^ during the maximal growing season (in AMJ 2022).

This box model approach yields flux estimates that are similar to those in previous studies. The derived annual daytime average fossil fuel CO_2_ flux of 19.7 ± 0.9 μmol m^–2^ s^–1^ is consistent with the adjusted Hestia-LA emissions of 23.2 μmol m^–2^ s^–1^ (see red dashed line in Figure 7) within the 2σ uncertainty bounds of 4.8 μmol m^–2^ s^–1^ on the box model inference (see section 3.2). Hestia-LA emissions for 2015 are modified to 2022 using the CARB greenhouse gas emission inventory estimated for California. While seasonal daytime fossil fuel CO_2_ flux varies between 22.3 and 23.9 μmol m^–2^ s^–1^ in adjusted Hestia-LA emissions, we observe variation between 8.0 and 20.9 μmol m^–2^ s^–1^. Asimow et al.^15^ also observed large seasonality in fossil fuel CO_2_ emissions in the San Francisco Bay Area and attributed the variation to a seasonal cycle in natural gas use. The maximum negative daytime biogenic flux that we observed is −6.7 ± 0.7 μmol m^–2^ s^–1^ in AMJ 2022. Note that the 1σ uncertainty in seasonal daytime biogenic flux derived in section 3.2 is 5.4 μmol m^–2^ s^–1^ (see the green dashed line in Figure 6). Recently developed biogenic models, estimating biogenic fluxes from vegetation remote sensing data, also reported negative fluxes of a similar magnitude from 0 to −15 μmol m^–2^ s^–1^ during the growing season for various cities in the United States, including LA.^45,46^

We combine observations from a dense sensor network with a box model for quantifying CO_2_ and CO emissions. The approach is simpler compared to computationally intense inverse methods and could be easily applied to other gases, such as NO_*x*_, O_3_, and aerosols. However, uncertainties caused by various assumptions made in the box model approach as well as uncertainties in each variable needed to quantify emissions propagate to the overall uncertainty. Furthermore, this method strongly depends upon the value for the effective mixing height that could result in a systematic bias in the flux estimates. We suggest using synthetic data experiments to derive an appropriate effective mixing height to minimize the systematic error. We have derived a constant scaling factor to estimate the effective mixing height for the daytime; however, this could be improved using various scaling factors for different times of day or different atmospheric conditions.

We apply this approach to CO and CO_2_ observations independently and then combine information from the two species with the ratio of COxs and CO_2_ff obtained from flask measurements of ^14^CO_2_ and CO collected in LA. This enables us to partition total CO_2_ emissions into fossil fuel and biogenic emissions that show good agreement with their known patterns and bottom-up emission estimates. We find the diurnal patterns in fossil fuel and biogenic flux as expected, showing larger fossil fuel emissions and larger biogenic update during the daytime. The seasonal variation in biogenic emissions as determined in our model corresponds to EVI observations, and the seasonal variation in fossil fuel emissions agrees well with previous studies. Lastly, derived annual daytime flux estimates match the fluxes from bottom-up fossil fuel emission inventory and biogenic models, providing additional support for this approach. We show that the biosphere can consume up to 60% of fossil fuel emissions in the growing season during the daytime. Nighttime flux estimates can be improved by finding effective mixing heights and a ratio of COxs and CO_2_ff suitable for early morning and nighttime.

We used this first year of observations to describe seasonal variation. We look forward to assessing long-term emission trends of CO_2_ and other pollutants not only here in LA but also in other cities, such as Providence, RI, and Glasgow, Scotland, where BEACO_2_N sensors have recently been installed. Additionally, we aim to extend our analysis to encompass a broader network, exploring regional differences by grouping sites according to their locations.

## Supporting Information Available

Detailed description of SUNVEx-LA flask measurements, including the estimation of CO_2_ ff and COxs from these measurements, as well as a synthetic data experiment to determine the effective mixing height using a simplified emission inventory and bottom-up CO_2_ emission, CO emission, and *R* (CO/CO_2_ ratio) estimates for each source sector to calculate *R*_ff_ for LA basin (PDF)

## References

[ref1] United Nations Framework Convention on Climate Change (UNFCCC). Paris Agreement. 21st Conference of Parties (COP21); Paris, France, Dec 12, 2015.

[ref2] Yu K. A.; McDonald B. C.; Harley R. A. Evaluation of Nitrogen Oxide Emission Inventories and Trends for On-Road Gasoline and Diesel Vehicles. Environ. Sci. Technol. 2021, 55, 6655 10.1021/acs.est.1c00586. 33951912

[ref3] McDonald B. C.; McBride Z. C.; Martin E. W.; Harley R. A. High-resolution Mapping of Motor Vehicle Carbon Dioxide Emissions. J. Geophys. Res. Atmos. 2014, 119 (9), 5283–5298. 10.1002/2013JD021219.

[ref4] Gurney K. R.; Patarasuk R.; Liang J.; Song Y.; O’Keeffe D.; Rao P.; Whetstone J. R.; Duren R. M.; Eldering A.; Miller C. E. The Hestia Fossil Fuel CO_2_ Emissions Data Product for the Los Angeles Megacity (Hestia-LA). Earth Syst. Sci. Data 2019, 11, 1309–1335. 10.5194/essd-11-1309-2019.

[ref5] Gately C. K.; Hutyra L. R.; Peterson S.; Sue Wing I. Urban Emissions Hotspots: Quantifying Vehicle Congestion and Air Pollution Using Mobile Phone GPS Data. Environ. Pollut. 2017, 229, 496–504. 10.1016/j.envpol.2017.05.091. 28628865

[ref6] Lauvaux T.; Miles N. L.; Deng A.; Richardson S. J.; Cambaliza M. O.; Davis K. J.; Gaudet B.; Gurney K. R.; Huang J.; O’Keefe D.; Song Y.; Karion A.; Oda T.; Patarasuk R.; Razlivanov I.; Sarmiento D.; Shepson P.; Sweeney C.; Turnbull J. C.; Wu K. High-Resolution Atmospheric Inversion of Urban CO_2_ Emissions during the Dormant Season of the Indianapolis Flux Experiment (INFLUX). J. Geophys. Res. 2016, 121 (10), 5213–5236. 10.1002/2015JD024473. PMC7430513 32818124

[ref7] Turnbull J. C.; Karion A.; Davis K. J.; Lauvaux T.; Miles N. L.; Richardson S. J.; Sweeney C.; McKain K.; Lehman S. J.; Gurney K. R.; Patarasuk R.; Liang J.; Shepson P. B.; Heimburger A.; Harvey R.; Whetstone J. Synthesis of Urban CO_2_ Emission Estimates from Multiple Methods from the Indianapolis Flux Project (INFLUX). Environ. Sci. Technol. 2019, 53 (1), 287–295. 10.1021/acs.est.8b05552. 30520634

[ref8] Sargent M.; Barrera Y.; Nehrkorn T.; Hutyra L. R.; Gately C. K.; Jones T.; McKain K.; Sweeney C.; Hegarty J.; Hardiman B.; Wang J. A.; Wofsy S. C. Anthropogenic and Biogenic CO_2_ Fluxes in the Boston Urban Region. Proc. Natl. Acad. Sci. U. S. A. 2018, 115 (29), 7491–7496. 10.1073/pnas.1803715115. 29967154 PMC6055148

[ref9] Turner A. J.; Kim J.; Fitzmaurice H.; Newman C.; Worthington K.; Chan K.; Wooldridge P. J.; Köehler P.; Frankenberg C.; Cohen R. C. Observed Impacts of COVID-19 on Urban CO_2_ Emissions. Geophys. Res. Lett. 2020, 47, e2020GL090037 10.1029/2020GL090037.

[ref10] Lauvaux T.; Gurney K. R.; Miles N. L.; Davis K. J.; Richardson S. J.; Deng A.; Nathan B. J.; Oda T.; Wang J. A.; Hutyra L.; Turnbull J. C. Policy-Relevant Assessment of Urban CO_2_ emissions. Environ. Sci. Technol. 2020, 54 (16), 10237–10245. 10.1021/acs.est.0c00343. 32806908

[ref11] Roten D.; Lin J. C.; Das S.; Kort E. A. Constraining Sector-Specific CO_2_ Fluxes Using Space-Based XCO_2_ Observations Over the Los Angeles Basin. Geophys. Res. Lett. 2023, 50 (21), 1–11. 10.1029/2023GL104376.

[ref12] Ye X.; Lauvaux T.; Kort E. A.; Oda T.; Feng S.; Lin J. C.; Yang E. G.; Wu D. Constraining Fossil Fuel CO_2_ Emissions From Urban Area Using OCO-2 Observations of Total Column CO_2_. J. Geophys. Res. Atmos. 2020, 125 (8), 1–29. 10.1029/2019JD030528.

[ref13] Turner A. J.; Shusterman A. A.; McDonald B. C.; Teige V.; Harley R. A.; Cohen R. C. Network Design for Quantifying Urban CO_2_ Emissions: Assessing Trade-Offs between Precision and Network Density. Atmos. Chem. Phys. 2016, 16, 13465–13475. 10.5194/acp-16-13465-2016.

[ref14] Fitzmaurice H. L.; Turner A. J.; Kim J.; Chan K.; Delaria E. R.; Newman C.; Wooldridge P.; Cohen R. C. Assessing Vehicle Fuel Efficiency Using a Dense Network of CO_2_ Observations. Atmos. Chem. Phys. 2022, 22 (6), 3891–3900. 10.5194/acp-22-3891-2022.

[ref15] Asimow N. G.; Turner A. J.; Cohen R. C. Sustained Reductions of Bay Area CO_2_ Emissions 2018–2022. Environ. Sci. Technol. 2024, 58 (15), 6586–6594. 10.1021/acs.est.3c09642. 38572839 PMC11025126

[ref16] Shusterman A. A.; Kim J.; Lieschke K. J.; Newman C.; Wooldridge P. J.; Cohen R. C. Observing Local CO_2_ Sources Using Low-Cost, near-Surface Urban Monitors. Atmos. Chem. Phys. 2018, 18 (18), 13773–13785. 10.5194/acp-18-13773-2018.

[ref17] Kim J.; Turner A. J.; Fitzmaurice H. L.; Delaria E. R.; Newman C.; Wooldridge P. J.; Cohen R. C. Observing Annual Trends in Vehicular CO_2_ Emissions. Environ. Sci. Technol. 2022, 56 (7), 3925–3931. 10.1021/acs.est.1c06828. 35324199

[ref18] Djuricin S.; Pataki D. E.; Xu X. A Comparison of Tracer Methods for Quantifying CO_2_ Sources in an Urban Region. J. Geophys. Res. 2010, 115 (14), 1–13. 10.1029/2009JD012236.

[ref19] Turnbull J. C.; Miller J. B.; Lehman S. J.; Tans P. P.; Sparks R. J.; Southon J. Comparison of ^14^CO_2_, CO, and SF_6_ as Tracers for Recently Added Fossil Fuel CO_2_ in the Atmosphere and Implications for Biological CO_2_ Exchange. Geophys. Res. Lett. 2006, 33, 2–6. 10.1029/2005GL024213.

[ref20] Newman S.; Jeong S.; Fischer M. L.; Xu X.; Haman C. L.; Lefer B.; Alvarez S.; Rappenglueck B.; Kort E. A.; Andrews A. E.; Peischl J.; Gurney K. R.; Miller C. E.; Yung Y. L. Diurnal Tracking of Anthropogenic CO_2_ Emissions in the Los Angeles Basin Megacity during Spring 2010. Atmos. Chem. Phys. 2013, 13 (8), 4359–4372. 10.5194/acp-13-4359-2013.

[ref21] Levin I.; Karstens U. Inferring High-Resolution Fossil Fuel CO_2_ Records at Continental Sites from Combined ^14^CO_2_ and CO Observations. Tellus, Ser. B Chem. Phys. Meteorol. 2022, 59 (2), 245–250. 10.1111/j.1600-0889.2006.00244.x.

[ref22] Verhulst K. R.; Karion A.; Kim J.; Salameh P. K.; Keeling R. F.; Newman S.; Miller J. B.; Sloop C.; Pongetti T.; Rao P.; Wong C.; Hopkins F. M.; Yadav V.; Weiss R. F.; Duren R. M.; Miller C. E. Carbon Dioxide and Methane Measurements from the Los Angeles Megacity Carbon Project—Part 1: Calibration, Urban Enhancements, and Uncertainty Estimates. Atmos. Chem. Phys. 2017, 17 (13), 8313–8341. 10.5194/acp-17-8313-2017. PMC6459414 30984251

[ref23] Kim J.; Verhulst K.; Verhulst K.; Salameh P.; Cox A.; Walker S.; Paplawsky B.; Prinzivalli S.; Fain C.; Stock M.; DiGangi E.; Biggs B.; Angel B.; Karion A.; Pongetti T.; Callahan W.; Weiss R. F.; Keeling R. F.; Miller C. E. In Situ Carbon Dioxide, Methane, and Carbon Monoxide Mole Fractions from the Los Angeles Megacity Carbon Project; National Institute of Standards and Technology (NIST): Gaithersburg, MD, 2021; 10.18434/mds2-2388.

[ref24] Shusterman A. A.; Teige V. E.; Turner A. J.; Newman C.; Kim J.; Cohen R. C. The BErkeley Atmospheric CO_2_ Observation Network: Initial Evaluation. Atmos. Chem. Phys. 2016, 16, 13449–13463. 10.5194/acp-16-13449-2016.

[ref25] Kim J.; Shusterman A. A.; Lieschke K. J.; Newman C.; Cohen R. C. The Berkeley Atmospheric CO_2_ Observation Network: Field Calibration and Evaluation of Low-Cost Air Quality Sensors. Atmos. Meas. Technol. 2018, 11 (4), 1937–1946. 10.5194/amt-11-1937-2018.

[ref26] Delaria E. R.; Kim J.; Fitzmaurice H. L.; Newman C.; Wooldridge P. J.; Worthington K.; Cohen R. C. The Berkeley Environmental Air-Quality and CO_2_ Network: Field Calibrations of Sensor Temperature Dependence and Assessment of Network Scale CO_2_ Accuracy. Atmos. Meas. Technol. 2021, 14 (8), 5487–5500. 10.5194/amt-14-5487-2021.

[ref27] Patel M. Y.; Vannucci P. F.; Kim J.; Berelson W. M.; Cohen R. C. Towards a Universal Hygroscopic Growth Calibration for Low-Cost PM_2.5_ Sensors. EGUsphere 2023, 10.5194/egusphere-2023-1701.

[ref28] Strong C.; Stwertka C.; Bowling D. R.; Stephens B. B.; Ehleringer J. R. Urban Carbon Dioxide Cycles within the Salt Lake Valley: A Multiple-Box Model Validated by Observations. J. Geophys. Res. Atmos. 2011, 116 (15), 1–12. 10.1029/2011JD015693.

[ref29] Balashov N. V.; Davis K. J.; Miles N. L.; Lauvaux T.; Richardson S. J.; Barkley Z. R.; Bonin T. A. Background Heterogeneity and Other Uncertainties in Estimating Urban Methane Flux: Results from the Indianapolis Flux Experiment (INFLUX). Atmos. Chem. Phys. 2020, 20 (7), 4545–4559. 10.5194/acp-20-4545-2020.

[ref30] Turnbull J. C.; Sweeney C.; Karion A.; Newberger T.; Lehman S. J.; Tans P. P.; Davis K. J.; Lauvaux T.; Miles N. L.; Richardson S. J.; Cambaliza M. O.; Shepson P. B.; Gurney K. R.; Patarasuk R.; Razlivanov I. Toward Quantification and Source Sector Identification of Fossil Fuel CO_2_ Emissions from an Urban Area: Results from the INFLUX Experiment. J. Geophys. Res. 2015, 120 (1), 292–312. 10.1002/2014JD022555.

[ref31] Vogel F. R.; Hammer S.; Steinhof A.; Kromer B.; Levin I. Implication of Weekly and Diurnal ^14^C Calibration on Hourly Estimates of CO-Based Fossil Fuel CO_2_ at a Moderately Polluted Site in Southwestern Germany. Tellus, Ser. B Chem. Phys. Meteorol. 2022, 62 (5), 512–520. 10.1111/j.1600-0889.2010.00477.x.

[ref32] Wu K.; Davis K. J.; Miles N. L.; Richardson S. J.; Lauvaux T.; Sarmiento D. P.; Balashov N. V.; Keller K.; Turnbull J. C.; Gurney K. R.; Liang J.; Roest G. Source Decomposition of Eddy-Covariance CO_2_ flux Measurements for Evaluating a High-Resolution Urban CO_2_ emissions Inventory. Environ. Res. Lett. 2022, 17 (7), 074035 10.1088/1748-9326/ac7c29.

[ref33] Vimont I. J.; Turnbull J. C.; Petrenko V. V.; Place P. F.; Sweeney C.; Miles N. L.; Richardson S.; Vaughn B. H.; White J. W. C. An Improved Estimate for the δ^13^C and δ^18^O Signatures of Carbon Monoxide Produced from Atmospheric Oxidation of Volatile Organic Compounds. Atmos. Chem. Phys. 2019, 19 (13), 8547–8562. 10.5194/acp-19-8547-2019.

[ref34] Griffin R. J.; Chen J.; Carmody K.; Vutukuru S.; Dabdub D. Contribution of Gas Phase Oxidation of Volatile Organic Compounds to Atmospheric Carbon Monoxide Levels in Two Areas of the United States. J. Geophys. Res. Atmos. 2007, 112 (10), 1–19. 10.1029/2006JD007602.

[ref35] Miller J. B.; Lehman S. J.; Verhulst K. R.; Miller C. E.; Duren R. M.; Yadav V.; Newman S.; Sloop C. D. Large and Seasonally Varying Biospheric CO_2_ Fluxes in the Los Angeles Megacity Revealed by Atmospheric Radiocarbon. Proc. Natl. Acad. Sci. U. S. A. 2020, 117 (43), 26681–26687. 10.1073/pnas.2005253117. 33046637 PMC7604494

[ref36] Lin J. C.; Gerbig C.; Wofsy S. C.; Andrews A. E.; Daube B. C.; Davis K. J.; Grainger C. A. A Near-Field Tool for Simulating the Upstream Influence of Atmospheric Observations: The Stochastic Time-Inverted Lagrangian Transport (STILT) Model. J. Geophys. Res. Atmos. 2003, 108 (D16), 4493 10.1029/2002JD003161.

[ref37] Gerbig C.; Lin J. C.; Wofsy S. C.; Daube B. C.; Andrews A. E.; Stephens B. B.; Bakwin P. S.; Grainger C. A. Toward Constraining Regional-Scale Fluxes of CO_2_ with Atmospheric Observations over a Continent: 2. Analysis of COBRA Data Using a Receptor-Oriented Framework. J. Geophys. Res. Atmos. 2003, 108 (D24), 4757-ACH6 10.1029/2003JD003770.

[ref38] Gaudet B. J.; Lauvaux T.; Deng A.; Davis K. J. Exploration of the Impact of Nearby Sources on Urban Atmospheric Inversions Using Large Eddy Simulation. Elementa 2017, 5, 60 10.1525/elementa.247.

[ref200] Verreyken B. W. D., Top-down Evaluation of Volatile Chemical Production Emissions using a Lagrangian Framework. *Environmental Science and Technology* 2024, in review. 10.1021/acs.est.4c10117 40176711

[ref39] Lian J.; Lauvaux T.; Utard H.; Bréon F. M.; Broquet G.; Ramonet M.; Laurent O.; Albarus I.; Chariot M.; Kotthaus S.; Haeffelin M.; Sanchez O.; Perrussel O.; Denier Van Der Gon H. A.; Dellaert S. N. C.; Ciais P. Can We Use Atmospheric CO_2_ Measurements to Verify Emission Trends Reported by Cities? Lessons from a 6-Year Atmospheric Inversion over Paris. Atmos. Chem. Phys. 2023, 23 (15), 8823–8835. 10.5194/acp-23-8823-2023.

[ref40] Newman S.; Xu X.; Gurney K. R.; Hsu Y. K.; Li K. F.; Jiang X.; Keeling R. F.; Feng S.; O’Keefe D.; Patarasuk R.; Wong K. W.; Rao P.; Fischer M. L.; Yung Y. L. Toward Consistency between Trends in Bottom-up CO_2_ Emissions and Top-down Atmospheric Measurements in the Los Angeles Megacity. Atmos. Chem. Phys. 2016, 16 (6), 3843–3863. 10.5194/acp-16-3843-2016.

[ref41] He L.; Zeng Z.; Pongetti T. J.; Wong C.; Liang J.; Gurney K. R.; Newman S.; Yadav V.; Verhulst K. R.; Miller C. E.; Duren R.; Frankenberg C.; Wennberg P. O.; Shia R.; Yung Y. L.; Sander S. P. Atmospheric Methane Emissions Correlate With Natural Gas Consumption From Residential and Commercial Sectors in Los Angeles. Geophys. Res. Lett. 2019, 46 (14), 8563–8571. 10.1029/2019GL083400.

[ref42] Zimmerman N.; Li H. Z.; Ellis A.; Hauryliuk A.; Robinson E. S.; Gu P.; Shah R. U.; Ye Q.; Snell L.; Subramanian R.; Robinson A. L.; Apte J. S.; Presto A. A. Improving Correlations between Land Use and Air Pollutant Concentrations Using Wavelet Analysis: Insights from a Low-Cost Sensor Network. Aerosol Air Qual. Res. 2020, 20 (2), 314–328. 10.4209/aaqr.2019.03.0124.

[ref43] Yadav V.; Ghosh S.; Mueller K.; Karion A.; Roest G.; Gourdji S. M.; Lopez-Coto I.; Gurney K. R.; Parazoo N.; Verhulst K. R.; Kim J.; Prinzivalli S.; Fain C.; Nehrkorn T.; Mountain M.; Keeling R. F.; Weiss R. F.; Duren R.; Miller C. E.; Whetstone J. The Impact of COVID-19 on CO_2_ Emissions in the Los Angeles and Washington DC/Baltimore Metropolitan Areas. Geophys. Res. Lett. 2021, 48 (11), 1–10. 10.1029/2021GL092744. PMC8206775 34149111

[ref44] Schaaf C. B.; Gao F.; Strahler A. H.; Lucht W.; Li X.; Tsang T.; Strugnell N. C.; Zhang X.; Jin Y.; Muller J.-P.; Lewis P.; Barnsley M.; Hobson P.; Disney M.; Roberts G.; Dunderdale M.; Doll C.; D’Entremont R. P.; Hu B.; Liang S.; Privette J. L.; Roy D. First Operational BRDF, Albedo Nadir Reflectance Products from MODIS. Remote Sens. Environ. 2002, 83 (1–2), 135–148. 10.1016/S0034-4257(02)00091-3.

[ref45] Wu D.; Lin J. C.; Duarte H. F.; Yadav V.; Parazoo N. C.; Oda T.; Kort E. A. A Model for Urban Biogenic CO_2_ Fluxes: Solar-Induced Fluorescence for Modeling Urban Biogenic Fluxes (SMUrF v1). Geosci. Model Dev. 2021, 14 (6), 3633–3661. 10.5194/gmd-14-3633-2021.

[ref46] Wei D.; Reinmann A.; Schiferl L. D.; Commane R. High Resolution Modeling of Vegetation Reveals Large Summertime Biogenic CO_2_ Fluxes in New York City. Environ. Res. Lett. 2022, 17 (12), 124031 10.1088/1748-9326/aca68f.

