# Supplementary material for: Observing Anthropogenic and Biogenic CO2 Emissions in Los Angeles Using a Dense Sensor Network
Source: Environ Sci Technol. 2025 Feb 13;59(7):3508–17. doi: 10.1021/acs.est.4c11392 (PMC11866928; doi:10.1021/acs.est.4c11392)
Supplement: Supplementary file 1 — es4c11392_si_001.pdf [file es4c11392_si_001.pdf]

Supporting Information for:

Observing anthropogenic and biogenic CO_2 emissions in Los Angeles using a dense sensor network

Jinsol Kim[1*], William M. Berelson[1], Nick Everett Rollins[1], Naomi G. Asimow[2], Catherine Newman[3], Ronald C. Cohen[2,3], John B. Miller[4], Brian C. McDonald[5], Jeff Peischl[5,6], and Scott J. Lehman[7]

[1]Department of Earth Science, University of Southern California, Los Angeles, CA 90089, USA
[2]Department of Earth and Planetary Science, University of California, Berkeley, Berkeley, CA 94720, USA
[3]Department of Chemistry, University of California, Berkeley, Berkeley, CA 94720, USA
[4]National Oceanic and Atmospheric Administration Global Monitoring Laboratory, Boulder, CO 80305, USA
[5]National Oceanic and Atmospheric Administration Chemical Sciences Laboratory, Boulder, CO 80305, USA
[6]Cooperative Institute for Research in Environmental Sciences, University of Colorado Boulder, Boulder, CO 80309, USA
[7]Institute of Arctic and Alpine Research, University of Colorado, Boulder, CO 80309, USA

[*]*Correspondence to* Jinsol Kim (jinsolki@usc.edu)

This 9-page PDF file includes:

Supplementary text S1 to S2
Figures S1 to S3
Tables S1
SI References

S1. SUNVEx-LA flask measurements

During SUNVEx-LA (from 8/5 to 9/3), flask air samples were collected every day at 2 p.m. local standard time at University of Southern California, California State University, Fullerton, and California Institute of Technology. We use air samples collected at University of Southern California (USC; see Figure S3) using National Oceanic and Atmospheric Administration (NOAA) programmable flask packages (PFPs) and programmable compressor packages (Sweeney et al. 2015). The collected samples were transported to the NOAA Global Monitoring Laboratory for analysis of greenhouse gases, including CO_2 and CO, using NOAA's highly precise and accurate measurement system for greenhouse gases (Sweeney et al., 2015). Following the initial measurement, residual air from PFP flasks was extracted, and CO_2 was isolated for ^{14}C measurement using well-established cryogenic and mass spectrometric techniques (Lehman et al. 2013). $\delta^{13}CO_2$ is measured by dual inlet isotope ratio mass spectrometry at the INSTAAR Stable Isotope Laboratory (Sweeney et al. 2015; Vaughn et al. 2004) The samples were further processed at the University of Colorado, Boulder, Institute of Arctic and Alpine Research (INSTAAR), where they were purified, graphitized, and packed into individual targets. Finally, the samples were sent to the University of California, Irvine, Keck Accelerator Mass Spectrometry Facility for high-precision $\Delta^{14}C$ measurement.

1.1 CO_2ff and COxs estimation

CO_2ff is estimated based on mass balances for the atmospherically conserved quantities $\Delta^{14}C \times CO_2$ and CO_2 (e.g. Miller et al. 2020; Turnbull et al. 2006):

$$C_{ff} = \frac{C_{obs}(\Delta_{obs} - \Delta_{bkg})}{(\Delta_{ff} - \Delta_{bkg})} - \frac{C_r(\Delta_r - \Delta_{bkg})}{(\Delta_{ff} - \Delta_{bkg})} \quad (1)$$

Measured CO_2 mole fractions and $\Delta^{14}C$ values are abbreviated as C and Δ. Subscripts ‘obs’, ‘bkg’, ‘ff’ and ‘r’ represent observations, background, fossil fuel, and respiration, respectively. Δ_{ff} is equal to -1000 ‰. As in the Miller et al. (2020) study focusing on LA, we estimate the value of the small respiratory term, $-C_r(\Delta_r - \Delta_{bkg})/(\Delta_{ff} - \Delta_{bkg})$, as 0.25 ppm. Following Miller *et al.* (2020), we define backgrounds using nighttime (2 AM local standard time) measurements made at Mount Wilson Observatory (MWO). Obvious outliers corresponding to pollution events

indicated by anomalously elevated values were excluded and then interpolated to the time of observations by fitting curves to the screened MWO data.

During the latter half of the campaign, smokes from multiple Northern California wildfires inflowed into the Los Angeles area. In flask measurements, high CO concentration is observed from August 21st to the end of the campaign corresponding to the high CO emission rate of fires while CO_2 concentration stays low. CO background determined using MWO is relatively constant which results in increase in COxs (CO enhancement above background) and consequentially high ratio of COxs and CO_2ff. We exclude this period to achieve a realistic local value for the ratio of COxs and CO_2ff.

S2. A synthetic data experiment using uniform emissions across the LA basin

We construct an emission map with a uniform emission rate across the LA basin, ensuring that the emission rate follows the temporal variation in total emissions in the LA basin and that the simulated CO_2 corresponds to the magnitude of observed CO_2. We use the same footprints and background concentration described in section 2.4 to generate the synthetic enhancement from July 2021 to July 2022 at hourly resolution. We apply the box model approach to the generated synthetic observations quantifying the flux estimates. Following the analysis in section 3.1, we use mixing heights varying between 0.1 h_{HRRR} and 1.0 h_{HRRR}, and evaluate the estimated flux compared to the uniform modeled flux to determine effective mixing height in the study region. We find an effective mixing height of 0.4 h_{HRRR}, consistent with results from a synthetic data experiment using the high-resolution fossil fuel emission product Hestia-LA (see Figure S3). Annual afternoon flux of the uniform emission rate is 20.9 μmol m^{-2} s^{-1} while Hestia-LA is 25.7 μmol m^{-2} s^{-1}. Uncertainty caused by the various assumptions made in the box model approach is quantified as ± 1.1 μmol m^{-2} s^{-1} (5 %) while more realistic synthetic data experiment using Hestia-LA reported ± 0.7 μmol m^{-2} s^{-1} (2 %).

Reference

Gurney, K. R., Risa Patarasuk, Jianming Liang, Yang Song, Darragh O'Keeffe, Preeti Rao, James R. Whetstone, Riley M. Duren, Annmarie Eldering, and Charles E. Miller. 2019. "The Hestia Fossil Fuel CO2 Emissions Data Product for the Los Angeles Megacity

(Hestia-LA).” *Earth System Science Data* 11:1309–35.

Kim, Jinsol, John B. Miller, Charles E. Miller, Scott J. Lehman, Sylvia E. Michel, Nick E. Rollins, and William M. Berelson. 2023. “Quantification of Fossil Fuel CO 2 from Combined CO , d 13 CO 2 and D 14 CO 2 Observations.” (June):1–25.

Lehman, Scott J., John B. Miller, Chad Wolak, John Southon, Pieter P. Tans, Stephen A. Montzka, Colm Sweeney, Arlyn Andrews, Brian LaFranchi, Thomas P. Guilderson, and J. C. Turnbull. 2013. “Allocation of Terrestrial Carbon Sources Using 14 CO 2 : Methods, Measurement, and Modeling.” *Radiocarbon* 55(3).

Miller, John B., Scott J. Lehman, Kristal R. Verhulst, Charles E. Miller, Riley M. Duren, Vineet Yadav, Sally Newman, and Christopher D. Sloop. 2020. “Large and Seasonally Varying Biospheric CO2 Fluxes in the Los Angeles Megacity Revealed by Atmospheric Radiocarbon.” *Proceedings of the National Academy of Sciences of the United States of America* 117(43):26681–87.

Newman, Sally, Xiaomei Xu, Hagit P. Affek, Edward Stolper, and Samuel Epstein. 2008. “Changes in Mixing Ratio and Isotopic Composition of CO2 in Urban Air from the Los Angeles Basin, California, between 1972 and 2003.” *Journal of Geophysical Research Atmospheres* 113(23):1–15.

Sweeney, Colm, Anna Karion, Sonja Wolter, Timothy Newberger, Doug Guenther, Jack A. Higgs, Arlyn Elyzabeth Andrews, Patricia M. Lang, Don Neff, Edward Dlugokencky, John B. Miller, Stephen A. Montzka, Ben R. Miller, Ken Alan Masarie, Sebastien Christophe Biraud, Paul C. Novelli, Molly Crotwell, Andrew M. Crotwell, Kirk Thoning, and Pieter P. Tans. 2015. “Seasonal Climatology of CO2 across North America from Aircraft Measurements in the NOAA/ESRL Global Greenhouse Gas Reference Network.” *Journal of Geophysical Research : Atmospheres* 5155–90.

Turnbull, J. C., John B. Miller, Scott J. Lehman, P. P. Tans, R. J. Sparks, and J. Southon. 2006. “Comparison of 14 CO2, CO, and SF6 as Tracers for Recently Added Fossil Fuel CO2 in the Atmosphere and Implications for Biological CO2 Exchange.” *Geophysical Research Letters* 33:2–6.

Vaughn, B. H., John B. Miller, D. F. Ferretti, and J. W. C. White. 2004. “Stable Isotope Measurements of Atmospheric CO2 and CH4.” in *Handbook of Stable Isotope Analytical Techniques*.

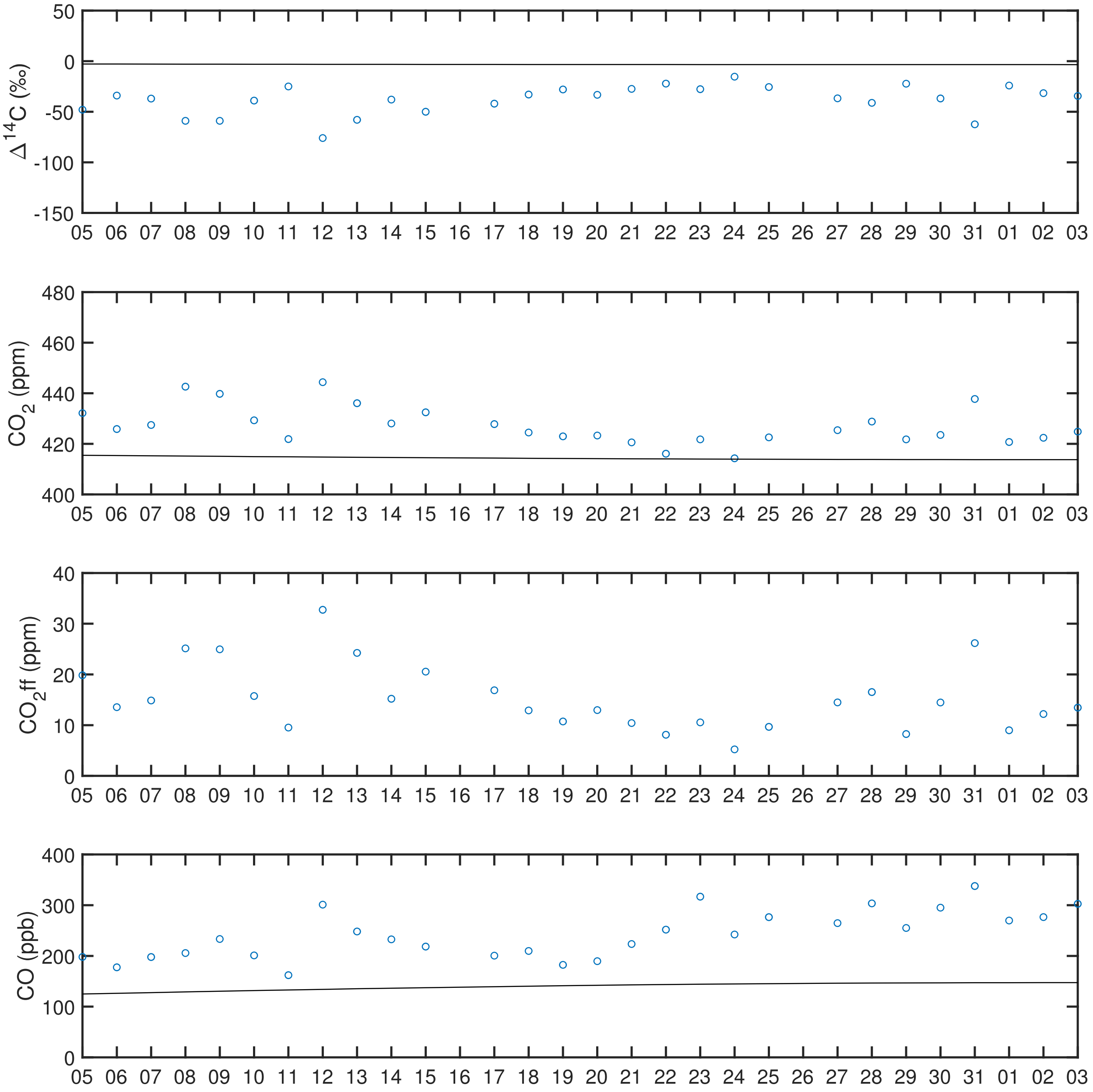

Figure S1. Timeseries of $\Delta^{14}C$, CO_2, derived CO_2ff, and CO. Black line represents background values.

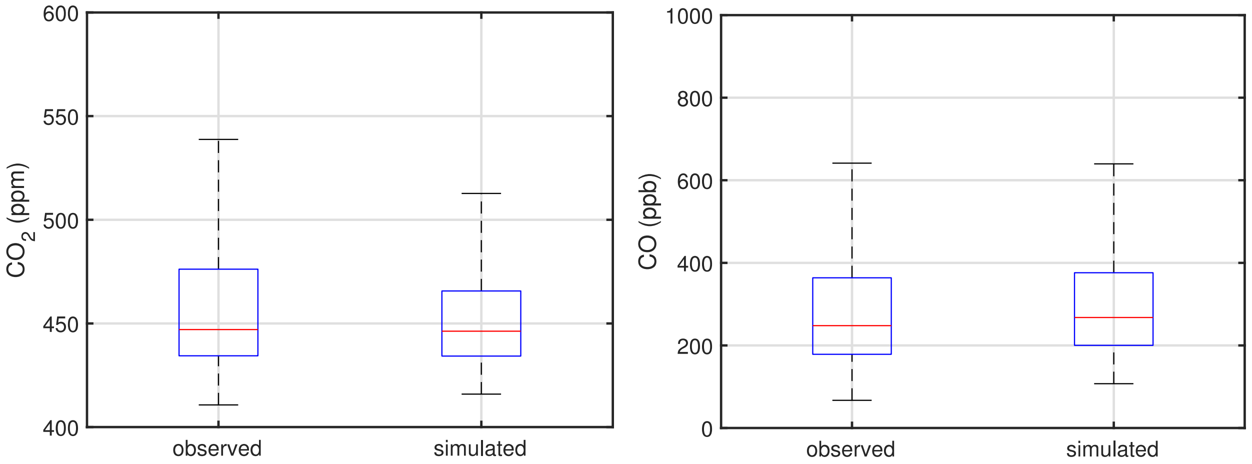

Figure S2. Boxplot of observed and simulated concentrations for CO2 (left) and CO (right). Note that simulated CO2 is from only fossil fuel CO2 emissions. Outliers are not displayed in the figure.

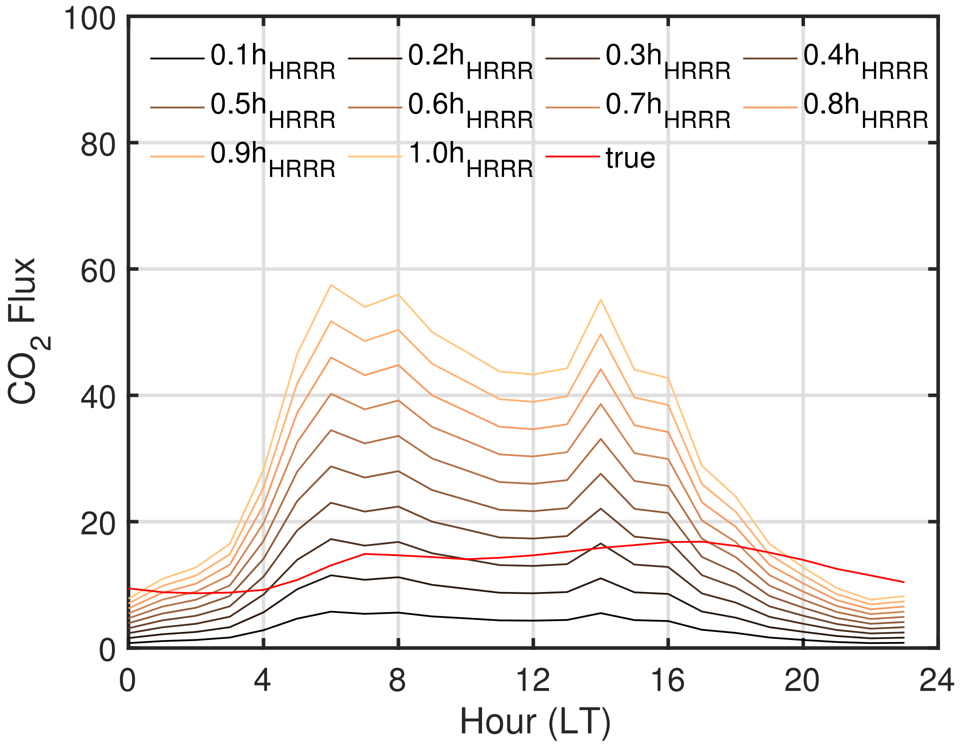

Figure S3. Diurnal pattern of fossil fuel CO_2 fluxes estimated from synthetic observation between July 2021 and July 2022 using effective mixing height varying between 0.1 h_{HRRR} and 1.0 h_{HRRR}, where h_{HRRR} is PBL height estimates from HRRR. Red line represents the modeled flux constructed to have uniform emission rate across the LA basin and used to generate the synthetic observations.

Table S1. Bottom-up CO_2 emission, CO emission, and R (CO/CO_2 ratio) estimates for each source sector for LA basin in 2019.

	CO_2 (MtC)	CO (MtC)	R = CO/CO_2 (ppb ppm^{-1})
Residential	1.83	0.009	2.16
Commercial	1.80	0.004	1.11
Industrial	7.00	0.013	0.77
Electricity Production	2.29	<0.001	0.17
On-road	11.17	0.147	5.65
Non-road	1.00	0.177	75.92
Airport	0.47	0.009	7.81
Rail	0.08	<0.001	2.96
CMV	0.44	0.009	8.83
**Total**	**26.08**	**0.370**	**6.08**
